# A Tool to Encourage Minimum Reporting Guideline Uptake for Data Analysis in Metabolomics

**DOI:** 10.3390/metabo9030043

**Published:** 2019-03-05

**Authors:** Elizabeth C. Considine, Reza M. Salek

**Affiliations:** 1 The Irish Centre for Fetal and Neonatal Translational Research (INFANT), Department of Obstetrics and Gynaecology, University College Cork, T12 YE02 Cork, Ireland; 2 The International Agency for Research on Cancer (IARC), 150 Cours Albert Thomas, 69372 Lyon CEDEX 08, France; SalekR@iarc.fr

**Keywords:** reproducibility, minimum guidelines, reporting, data analysis

## Abstract

Despite the proposal of minimum reporting guidelines for metabolomics over a decade ago, reporting on the data analysis step in metabolomics studies has been shown to be unclear and incomplete. Major omissions and a lack of logical flow render the data analysis’ sections in metabolomics studies impossible to follow, and therefore replicate or even imitate. Here, we propose possible reasons why the original reporting guidelines have had poor adherence and present an approach to improve their uptake. We present in this paper an R markdown reporting template file that guides the production of text and generates workflow diagrams based on user input. This R Markdown template contains, as an example in this instance, a set of minimum information requirements specifically for the data pre-treatment and data analysis section of biomarker discovery metabolomics studies, (gleaned directly from the original proposed guidelines by Goodacre at al). These minimum requirements are presented in the format of a questionnaire checklist in an R markdown template file. The R Markdown reporting template proposed here can be presented as a starting point to encourage the data analysis section of a metabolomics manuscript to have a more logical presentation and to contain enough information to be understandable and reusable. The idea is that these guidelines would be open to user feedback, modification and updating by the metabolomics community via GitHub.

## 1. Introduction

Metabolomics data mining describes the application of strategic data analysis methods incorporating artificial intelligence, machine learning, statistics and database operations to extract meaningful and useful information from high-dimensional and high-volume metabolomics datasets. This is a complex, time-consuming process including many steps with many possible options at each step. Further complexity is encountered in metabolomics investigations due to the fact that data analysis is often based on open source tools, each having their own parameter dependencies, also metabolomics datasets typically contain missing values and the handling of these can greatly influence the result of downstream analysis [1,2]. For detailed discussion and reviews of the data analysis step in metabolomics and its complexities, the reader is referred to the following publications [3,4,5,6,7].

Despite the obvious complexity and importance of the data mining step in the overall pipeline of any metabolomics study, this section of the workflow is often given scant attention in the write up of scientific research articles. Metabolomics data analysis sections have been found to be plagued by inconsistent reporting—specifically with regards to structure, details reported, and performance metrics used [3].

No standard method for how to analyse metabolomics data exists and therefore data analysis is in constant evolution, with new methods frequently being proposed in the literature. To discover the best methods, to build upon existing approaches and to conduct meta-analysis, the data analysis write up in metabolomics studies needs to be understandable and imitable, at a minimum. Furthermore, for those new to metabolomics data analysis, the starting point to construct a data analysis plan would most likely involve examining previously published research in the same field, with a view to reusing or adapting the various approaches. For these purposes, the current standard of reporting in the data analysis sections of metabolomics’ publications is woefully insufficient. The immediate improvement of reporting of the data analysis step is therefore vital to advance understanding and to promote reuses of data analysis protocols and eventually move closer to the ideal of reproducibility [8,9].

The metabolomics standards initiative (MSI) [10] was conceived in 2005, as an initiative of the Metabolomics Society. Under the MSI 5 working groups (WG) were established for each aspect of the metabolomic pipeline: biological context metadata WG, chemical analysis WG, data processing WG, ontology WG and data exchange WG; and a series of papers were published in 2007 with minimum reporting guidelines covering all areas of the metabolomics experiment [11,12,13,14,15]. Minimum reporting guidelines for data analysis in metabolomics [11] were first published in 2007. These comprehensive guidelines cover: 1: Design of Experiment (sample collection/matching, and data acquisition scheduling of samples); 2: Data Collection 3: Data pre-processing (data cleaning, outlier detection, row/column scaling, or other transformations; definition and parameterization of subsequent visualizations) 4: Data pre-treatment (row-wise and column-wise operations such as normalisation, scaling, centering and transformation to make data more amenable to statistical analysis); and finally, 5: Actual data analysis, which includes algorithm selection, univariate analysis and multivariate analysis. These reporting guidelines were published as an article, but were not subsequently published in the format of a guidelines checklist, with an explanation and elaboration document, nor were they formally disseminated. Results of our recent review on reporting the actual data analysis step in metabolomics indicate that these original reporting guidelines have had very poor take up, at least for the data pre-treatment and actual analysis section of metabolomics studies [2]. For example, 89% (23 out of a total of 25) of studies reviewed from the years 2008 to 2014 did not mention the proportion of missing values nor how the missing values were dealt with. Less than half the studies reviewed, reported on any kind of quality control procedure and less than half had any mention of outlier detection and/or removal.

Reasons why those original reporting guidelines for metabolomics have had such poor uptake may include the fact that they never progressed from the “proposed” stage to being formally published as practical guidelines along with a detailed “explanation and elaboration document” and they are not required by most journals for publishing a metabolomics manuscript. Also, their comprehensiveness may have inhibited their uptake, as there may have been an overwhelming amount of information to work through.

Strategies to increase the uptake and impact of guidelines can be adopted by their authors such as publishing the guidelines in multiple journals to ensure quicker and wider dissemination; also authors can approach journals and ask them to include the guidelines in their “Instructions to authors” section and publish commentaries to endorse them [16]. Official society and community wide recommendations can also positively influence the uptake of guidelines. There are a number of recommendations on a dedicated page of the EQUATOR (Enhancing the QUAlity and Transparency Of health Research) Network website [17] on how to effectively disseminate your reporting guideline: http://www.equator-network.org/toolkits/developing-a-reporting-guideline/disseminating-your-reporting-guideline/.) This would of course be in addition to previous recommendations of a supplementary audit, open code and script sharing [18].

In recognition of the complexity of data analysis in metabolomics, we propose that distinct reporting guidelines be drawn up for separate sections of the data analysis pipeline (design of experiment, data reduction and deconvolution, data pre-processing, data annotation and identification, data pre-treatment and data analysis) as their various steps are often carried out at different times, (sometimes years pass between different steps), by different individuals or groups of various skill sets, or even often outsourced to different locations. Adherence to the guidelines is more likely to be achieved by discretising each section of the pipeline into succinct guideline sets (modules), which can be adopted by the relevant analyst(s) at the time of manuscript writing and incorporated into the report.

In other established omics reporting guidelines’, namely MIAME [19] for microarrays and MIAPE [20] for proteomics, instructions do not exist for the data analysis part of the pipeline. The Equator (Enhancing the QUAlity and Transparency Of health Research) Network [17] is an international initiative aimed at promoting transparent and accurate reporting of health research studies to enhance the value and reliability of medical research literature. The Equator Network does not contain any guidelines for the reporting of multivariate data analysis/high dimensional data analysis/omics data analysis/supervised data analysis. The biosharing website MIBBI (Minimum Information for Biological and Biomedical Investigations) [21] does not contain any standards for data analysis reporting, but it does reference a standard called CIMR- Core Information for Metabolomics Reporting (CIMR) [22] which refers to the original proposed guidelines [11]. However, since these initial proposed guidelines in 2007, no further work has been published on the development or dissemination of metabolomics data analysis reporting guidelines. 

Since the Metabolomics Standards Initiative (MSI) [10], significant developments in data reporting standards in metabolomics have been made through many initiatives including COSMOS [23], MetaboLights [24] and FAIR [25], which endeavour to ensure the consistency of metadata between datasets, and facilitate data reuse and data mergers across studies [26]. However, with regards to the reporting of the data analysis of metabolomics studies, since the original guidelines [11] there have been no further advancements.

There has recently been a proliferation of reporting guidelines in biomedical research [27], there are currently 407 reporting guidelines on the Equator Network [17], many containing extensions and different versions. Despite this, compliance levels with these guidelines have been disappointing [28]. It has been noted that the “main problem” preventing the uptake of guidelines is that they are used too late in the research process, when it is too late to discover important things that have been missed or could have been done better [29]. The use of guidelines too late in the research process can be considered analogous to, and a part of a larger issue, where data analysis is frequently not being considered fully in the experimental design at the outset of a study [30].

### 1.1. Data Analysis Reporting Using R Markdown

With this information in mind, it is suggested that adherence to guidelines could be facilitated, if reporting guideline modules were contained in authoring tools such as the one presented in this study using R Markdown. R Markdown is a free and open source authoring framework [31]. R Markdown documents are fully reproducible and support dozens of static and dynamic output formats. A single R Markdown file can be used to both save and execute code and generate high quality reports that can be shared with an audience. R Markdown starts with a plain text file that is edited by the user that has the extension “.Rmd”. This plain text file then generates a new file that contains user selected text, code, and results from the .Rmd file. The new file can be a finished web page, PDF, MS Word document, slideshow, notebook, handout, book, dashboard, package vignette or other format. Besides R Markdown, there are other so called “digital notebooks” [32] that, in the vein of literate programming, support the connection of text to underlying code and analysis results [33]. For example Jupyter for R, Python and Julia (https://jupyter.org/) and matlabweb for MATLAB (https://ctan.org/pkg/matlabweb). 

As efforts towards computational reproducibility continue, an authoring tool in R Markdown such as the one presented here has the advantage that it can simultaneously achieve the aims of both ensuring reporting standards are adhered to, while also having the potential function as a digital notebook. Such an authoring tool could therefore ultimately provide an uninterrupted and transparent workflow from the initial stage of data in to the final output of an analysis report. The example of an authoring tool presented here, in this instance, is solely focused on reporting the statistical data analysis step of the pipeline, incorporating the data pre-treatment step and the actual data analysis step.

### 1.2. Objectives

1. To present a set of previously proposed minimum reporting guidelines in the form of a checklist specifically for the data analysis step of metabolomics biomarker discovery studies. There are typically four phases to this data analysis pipeline, although aside from pre-treatment, the other steps are not essential but are commonly used:
  - Data pre-treatment
  - Univariate data analysis to identify significant features that differ between groups
  - Multivariate data analysis
    ▪ Unsupervised data analysis to discover correlated features or identify hidden subgroups or to visualise separation and identify outliers
    ▪ Supervised data analysis, specifically for developing prediction models and/or biomarker identification.
  - Biomarker Candidate Performance Analysis
    ▪ Receiver Operating Curve (ROC) analysis
2. To provide an authoring tool to promote standardised comprehensive reporting on data analysis, which will also generate workflow diagrams.

## 2. Methods

### 2.1. Checklist of Minimum Information For Reporting Data Analysis In Metabolomics

The development of a reporting guideline checklist, specifically for the data pre-treatment and data analysis sections of the metabolomics pipeline, with a view to general applicability to other omic domains.

This checklist was compiled based on the complete information required to construct a workflow diagram and to repeat the analysis using the reader’s own version of code. The main areas of omissions, which we found lead to confusion and ambiguity when conducting our review [3], helped to inform this checklist.

Existing guidelines which helped to shape this guideline list, included the TRIPOD [34] statement, GRIPS [35] statement and REMARK guidelines [36]. Of course, the main document informing these guidelines is the original proposed guidelines for data analysis reporting in metabolomics by Goodacre et al. [11], which covers the reporting of every part of the data analysis of a metabolomics experiment from design of experiment through pre-processing to data pre-treatment and final analysis.

### 2.2. An Authoring Tool For Reporting Statistical Analysis Of Predictive Omics

The development of an authoring tool using R Markdown. This reporting guideline checklist is presented as a questionnaire in an R markdown file that guides the production of text and workflow diagrams based on user input. These reporting guidelines are intended to form a neutral and malleable framework and have general applicability and interoperability across various omics domains. These can be extended as needed by different domains or studies, but would represent a minimum set of information to be supplied whenever predictive data mining in metabolomics is carried out. We purposely do not develop a “user friendly” web interface, as the goal is for users to operate within the R Markdown environment.

## 3. Results

### 3.1. Minimum Information About A Data Analysis (MIDAS) Checklist (Guidelines Checklist Specifically for the Data Analysis Step)

Guidelines of two types generally exist: guidelines for reporting and guidelines for protocols. Since the area of data mining for metabolomics is still in rapid evolution, we suggest that guidelines or limitations on methodology at this point would be premature, as the optimal methods for extracting clinically useful biomarkers has clearly not been established. Therefore, these guidelines pertain only to reporting.

MIDAS Guidelines Checklist

Pre-treatment

- What are the dimensions of the dataset entering this phase of analysis?
- What percentage of the data is missing values?
- Is imputation (I) performed?
- If yes, describe the method.
- Is normalisation (N) performed?
- If yes, describe the method.
- Is transformation (T) performed?
- If yes, describe the method.
- Is scaling (S) performed?
- If yes, describe the method.
- Is filtering (F) applied to the dataset at this point?
- If yes, describe the method.
- Is a Quality Control/Quality Assessment (QC/QA) method employed on the dataset?
- Please describe.
- Outline the order of the pre-treatment steps performed on the dataset.
    E.g., I-> T-> S->N->F->QC
- Have the dimensions of the dataset changed from the outset of pre-treatment to the end of pre-treatment?
- Provide details on the package or program used for this phase of the analysis.
- If an in-house code is used, provide it or a link to it and also the language the code is written in.

Univariate analysis

- What are the dimensions of the dataset entering this phase of analysis?
- Is univariate testing performed?
- If yes, describe the method.
- Is a multiple testing correction employed with this method?
- If yes, describe the method.
- Are other methods of univariate testing performed?
- If yes, describe the methods.
- Are multiple testing correction employed with these methods?
- If yes, describe the method.
- Please report p-values and adjusted p-values.
- Please report test statistics and confidence intervals.
- Have the dimensions of the dataset changed from the outset of univariate analysis to the end of univariate analysis? If yes, provide the dimensions of the dataset at the end of univariate analysis and make it clear how the dimensions have changed.
- Provide details on the package or program used for this phase of the analysis.
- If in-house code is used, provide it or a link to it and also the language the code is written in.
- If a list of potential biomarkers is produced at this point, please state this explicitly.

Multivariate Analysis: Unsupervised analysis

- What are the dimensions of the dataset entering this phase of the analysis?
- Are unsupervised methods employed for visualisation and/ or data reduction and/or correlation analysis?
- If yes, describe the algorithm used.
- Is outlier detection and removal addressed at this point? If yes please describe and specify the outliers removed.
- Are unsupervised analysis methods used for clustering?
- If yes, describe and provide distance metric.
- Have the dimensions of the dataset changed? If yes, how and why?
- Provide the dimensions of the dataset at the end of unsupervised analysis.
- Provide details on the package or program used for this phase of the analysis.
- If in house code is used, provide it or a link to it and also the language the code is written in.
- If a list of potential biomarkers is produced at this point, please state this explicitly.

Multivariate Analysis: Supervised analysis

- What are the dimensions of the dataset at this point?
- Are supervised methods employed?
- If yes, describe the supervised analysis described fully enough to allow the imitation of the exact procedure. This would require reporting all the following information: all parameters; details of how data is split; details of how internal validation is conducted; details of how meta-parameter optimization is performed; details about the chosen metric for evaluating the performance of the classifier and finally the overall description of the workflow.
- Is more than one supervised method employed?
- If yes, describe the implementation of the other algorithm(s) fully enough to allow imitation of the exact procedure. This requires the reporting of all the following information: all parameters; details of how data is split; details of how internal validation is conducted; details of how meta-parameter optimization is performed; details about the chosen metric for evaluating the performance of the classifier and finally the overall description of the workflow.
- Is external validation employed?
- If yes, describe the source of external data. Is the data from the same location/ lab/timeline or a hold-out set from the original data?
- Provide a confusion matrix of results.
- Provide results as an average of n leave multiple-out and external predictions.
- Are potential biomarkers identified? If yes, list them.
- Have the dimensions of the dataset changed? If yes, how and why?
- Provide the dimensions of the dataset at the end of supervised analysis.
- Provide details on the package or program used for this phase of the analysis
- If in house code, is used provide it or a link to it and also the language the code is written in.
- If a list of potential biomarkers is produced at this point please state this explicitly.

Biomarker Performance Analysis:

Receiver Operating Curve (ROC) Analysis

- Is ROC analysis performed on the identified putative biomarkers?
- If yes, please report on AUC, sensitivity and specificity.
- Provide details on the package or program used for this phase of the analysis
- If in house code is used, provide it or a link to it and also the language the code is written in.

Another example of a facet of the data analysis report that could be developed by community participation and added to the existing template via the GitHub repository is the area of error analysis. The importance of error analysis in metabolomics and in particular the reporting of such error analysis has been advised by Moseley, who called for the reporting of “a detailed list of all known or potential biases and assumptions, along with results of any analysis and testing of these bias and assumptions; and results with adequate measures of uncertainty and confidence or at least a good explanation for why uncertainty and confidence measures are not provided” [37]. Moseley suggested to augment existing standards, in particular for reporting known and potential sources of bias, by borrowing from clinical standards like STARD [38] and CONSORT [39,40].

For other data analysis methods currently not covered here (for example, cluster analysis and other classification and feature selection methods), similar templates can be generated with their required parameter reporting and added to the existing templates via adding branches to the GitHub repository.

We actively invite the participation from metabolomics community users to become involved in this collaborative venture. Ideally, standards should be revisited on a regular schedule—preferably annually, to reflect the evolving knowledge and current best practices in the field.

We suggest uploading the R Markdown report document along with other raw and processed data as well the experimental metadata data during the submission of experimental metabolomics results to the relevant repository such as MetaboLights [41] or Metabolomics Workbench [42].

### 3.2. Link to GitHub Repository Containing Markdown Template

https://github.com/MSI-Metabolomics-Standards-Initiative/MIDAS

This R Markdown file containing the authoring template is contained in a GitHub repository. Having started as a code developer’s collaborative platform, GitHub [43] is now the largest online storage space of collaborative works that exists in the world, which makes it the ideal platform to share this R Markdown template file.

The beauty of R Markdown as stated above is that it can embed and execute code and this code can then be hidden or displayed in the final document. Even in the most basic of report writing templates, such as the one presented here, this aspect is very useful, as DiagrammeR [44], a flexible and powerful R package for generating graph and flowchart diagrams, can be used. It is necessary to state that because these diagrams depend on HTML and JavaScript for rendering, they can only be used in HTML based output formats (they do not work in PDFs or MS Word documents). This issue can be circumvented by can saving the workflow diagrams within ones preferred R Markdown tool such as RStudio as an image (JPEG, PNG, BMP, etc.) and inserting them into the final PDF or Word document version of the report.

The authoring tool presented here does not dictate or control the report produced by the data analyst. The report produced can continue to be edited after the final report is generated. It merely serves as a guide for the writer to construct their analysis report by reminding them of points to include. To our knowledge, this is the first instance that such a markdown tool has been proposed to aid and formalize reporting guideline uptake.

Continued community participation with a version-controlled set of standards is essential for the future development and adoption of any standards [45]. Once researchers recognise the benefits of sharing their data processing and data analysis approaches, the community can benefit from best practices in bioinformatics [18].

This authoring template is currently available on the Metabolomics Standards Initiative GitHub Repository and is open and welcoming to extension, modification and improvement from the metabolomics community and as such is considered a work in progress and a dynamic tool.

## 4. Discussion

Currently a copy-and paste paradigm, in which results are generated in a statistical package and copied and pasted to a report document, dominates data analysis reporting. Eventually, a complete move away from this antiquated copy-and-paste system, which is error prone and enables selective reporting, is needed in order to fulfill the requirements of reproducible research. However, in the meantime, in this instance, our version of the MIDAS reporting template allows users to manually input results that they have been obtained using other software, whilst also having the potential to contain fully executable code. This is so as not to exclude users other than R users from benefiting from using this reporting template as an authoring tool and to encourage the first steps towards reproducible research.

Employing R Markdown to help the uptake of minimum reporting standards goes further than providing a checklist. By encouraging scientists to consider reporting standards at the time of manuscript writing, it actively helps authors adhere to reporting guidelines. These guidelines should be viewed by authors as helpful to the writing process as opposed to a “yet another hurdle along the journey to publication” [27]. Also, requiring that parameter choices are revealed in reporting, even default ones from online data analysis tools, will encourage non-experts to deliberate on the choices they are making regarding the appropriate algorithms and parameters for the type of analysis that they are doing, which will further the advancement of the field. As mentioned earlier, such data analysis choices should ideally have been considered during the experimental design stage. Participation by the wider metabolomics community in updating and optimizing these guidelines could open the discussion on defining optimal performing data analysis workflows for specific research goals, depending on an individual study’s dataset limitations and the desired outcomes of the particular study.

We purposely do not provide a web-based user interface for accessing this R Markdown template, as we believe that data analysts need to become comfortable in environments such as R Markdown if the production of reproducible research papers is to become a reality. Furthermore, we feel that anyone who is capable of data analysis is more than capable of using R Markdown, without the need of a “user friendly” web interface.

A modularised system of authoring tools would encourage the uptake of guidelines on two fronts: The modularised facet will ensure that each researcher at each stage of the pipeline will be responsible for following the appropriate guidelines pertaining to their own area of expertise. The authoring tool part would ensure that guidelines are addressed at the time of writing up that particular section, as opposed to a set of rules to consider for application to the manuscript just before journal submission when the entire article has already been written.

Ideally, it is envisaged that such a modularised system of reporting guidelines’ authoring tools would evolve in the omics community, whereby these modules could be concatenated as needed depending on the experiment, each module corresponding to a stage in the workflow pipeline, and all modules being extensible and modifiable according to the domain and experiment in question.

In computational biology, extensibility and modifiability of tools are essential, so that new methods can develop and build on the old ones without repetition or reinventing the wheel. For this reason, this R Markdown file is not presented here as an end result, but is proposed as a starting point to encourage the data analysis section of metabolomics papers to have a more logical and stepwise presentation and to contain enough information to be understandable. So, even though this R Markdown file only attends to the authoring and not the analysis of metabolomics data, we hope that it will coax data analysts into the environment of R Markdown (and GitHub), and therefore be a nudge along the road towards readable, and ultimately, reproducible, metabolomics research.

Here are the instructions to use this R Markdown authoring template.

1. Go to the **GitHub** repository: https://github.com/MSI-Metabolomics-Standards-Initiative/MIDAS.
2. Click the “clone or download” button on the right hand side of the page and download the folder as a zip file.
3. Download latest version of R Studio if you do not have it.
4. Open the folder and open the *MIDAS.rmd* file in R studio.
5. Start editing and writing the report of your data analysis guided by the questions in green directly inside the *MIDAS.rmd* file.
6. After the pre-treatment, univariate analysis, multivariate analysis and error analysis sections have been completed the next section is to produce workflow diagrams.
7. Follow the instructions in green to produce a workflow diagram of pre-treatment steps.
8. Click on the knit button and knit to HTML to see how the generated report looks.
9. Knit to PDF or Word to render the report to a PDF or Word document as you wish.
10. PDF and Word reports will not contain the diagrams so these need to be saved in the viewer pane as an image (JPG /BMP etc) to your local folder.
11. Insert the workflow diagrams into your Word or PDF report that you have saved to your local folder.
12. Render the document to HTML and workflow diagrams will be included anyway.
